# Towards a comprehensive approach to mentalization-based treatment for children with autism: integrating attachment, neurosciences, and mentalizing

**DOI:** 10.3389/fpsyt.2023.1259432

**Published:** 2023-11-30

**Authors:** Stefanella Costa-Cordella, Patricia Soto-Icaza, Karin Borgeaud, Aitana Grasso-Cladera, Norka T. Malberg

**Affiliations:** ^1^Centro de Estudios en Psicología Clínica y Psicoterapia, Facultad de Psicología, Universidad Diego Portales, Santiago, Chile; ^2^Centro de Estudios en Neurociencia y Neuropsicología Humana, Facultad de Psicología, Universidad Diego Portales, Santiago, Chile; ^3^Millennium Institute for Depression and Personality Research (MIDAP), Santiago, Chile; ^4^Laboratorio de Neurociencia Social y Neuromodulación (neuroCICS), Centro de Investigación en Complejidad Social (CICS), Facultad de Gobierno, Universidad del Desarrollo, Santiago, Chile; ^5^Hospital Dr. Exequiel González Cortés, Santiago, Chile; ^6^Institute of Cognitive Science, Universität Osnabrück, Osnabrück, Germany; ^7^Yale Child Study Center, New Haven, CT, United States

**Keywords:** mentalization-based treatment, theory of mind, attachment, autism, neurodevelopment, neurodevelopmental disorders, child psychotherapy

## Abstract

Autism spectrum disorder (ASD) is diagnosed based on socio-communicative difficulties, which are believed to result from deficits in mentalizing, mainly evidenced by alterations in recognizing and responding to the mental states of others. In recent years, efforts have been made to develop mentalization-based treatment (MBT) models for this population. These models focus on enhancing individuals’ ability to understand and reflect on their own mental states, as well as those of others. However, MBT approaches for people with ASD are limited by their existing theoretical background, which lacks a strong foundation grounded in neuroscience-based evidence properly integrated with attachment, and mentalizing. These are crucial aspects for understanding psychological processes in autism, and as such, they play a pivotal role in shaping the development of tailored and effective therapeutic strategies for this specific population. In this paper we review evidence related to the neurobiological, interpersonal, and psychological dimensions of autism and their implications for mentalizing processes. We also review previous mentalization-based frameworks on the psychosis continuum to provide a comprehensive understanding of attachment, neurobiology, and mentalization domains in therapeutic approaches for autism. After presenting a synthesis of the literature, we offer a set of clinical strategies for the work with children with autism. Finally, we provide recommendations to advance the field towards more robust models that can serve as a basis for evidence-based therapeutic strategies.

## 1 Introduction

Autism spectrum disorder (ASD) is a neurodevelopmental disorder that affects communication and social interaction (1–4). Autism is a complex and heterogeneous condition that has undergone changes in its conceptualization and diagnostic criteria over the past few decades, shifting from a categorical to a dimensional perspective.

Individuals with ASD are often described as having difficulties in mentalizing - the ability to understand and think about other people’s thoughts, feelings, and intentions (5–13). Specifically, people on autistic spectrum may experience difficulties in mentalizing emotional states due to their struggles to understand social cues or interpret facial expressions accurately (5, 7, 14–18). This feature can lead to difficulties in interpersonal relationships and social functioning.

Therapeutic approaches to address these difficulties have arisen from mentalization-based treatment (MBT). While MBT was initially developed for individuals with borderline personality disorder (BPD), its application has broadened significantly over time. Today, it is used to treat a variety of psychological conditions in different populations, demonstrating its versatility and effectiveness as a therapeutic approach (19).

One of the MBT approach applications that might be interesting to look at when thinking about ASD is the development of MBT for individuals with psychosis (4, 20). Although individuals with ASD have different characteristics and challenges than individuals with BPD and psychosis, research has suggested an overlap between autistic and psychotic symptoms, particularly regarding problems with mentalizing or understanding the minds of others and oneself (4, 20–22). The psychosis spectrum is characterized by a rupture in the sense of “going-on-being” in the world, involving a diminished skill to relate with others, especially when it comes to emotional bonding (20). Therefore, these individuals experience mentalizing disruptions even in the premorbid and prodromal stages (4, 20). Nevertheless, mentalizing disruptions do not perform as aetiological or causal factors for the subsequent development of clinical psychosis. Instead, mentalizing capacities may be a protective factor (23) to mitigate early psychotic trajectory and foster recovery in individuals at high risk and clinically diagnosed with psychosis, respectively (20). In autism, similarly, deficits in mentalizing are not aetiological factors, but the ability to understand their own emotions and thoughts, as well as those of others, can serve as a protective factor that promotes resilience. In this way, enhancing and optimizing mentalizing could improve social functioning in autism. This would support the potential benefits of mentalization-based interventions in neurodevelopmental conditions like autism.

Considering this, we argue that interventions targeting mentalizing deficits could help alleviate psychological symptoms in individuals with ASD. MBT, with its focus on essential social cognitive processes that support social functioning, may particularly benefit individuals with ASD who struggle to identify and differentiate between different mental states in themselves and others. Furthermore, due to its emphasis on interpersonal relationships, MBT may assist individuals in managing socio-relational challenges.

In the following sections, we will review the evidence of social functioning difficulties in ASD from both symptomatological and neurobiological perspectives to provide a comprehensive understanding of the dimensions of ASD and their implications for mentalizing processes. We will also examine evidence related to MBTs and how insights from neuroscience research can inform the development of targeted interventions. Finally, we will propose clinical strategies that can be incorporated into existing mentalization-based models to better address the needs of children with ASD and present potential directions for advancing research in this field.

## 2 Social functioning and mentalizing in autism

From a clinical and neurocognitive perspective, behavioral difficulties in social functioning have been associated with autism from an early stage of development (6, 7, 14, 24). The Diagnostic and Statistical Manual of Mental Disorders, in its Fifth Edition (4) describes three main groups of a range of deficits in social communication and social interaction. First, deficits in social–emotional reciprocity, such as abnormal social approach and difficulties in maintaining two-way communication, decreased interest in shared interests, emotions, or affections, and failure to initiate or respond to social interactions. Secondly, impairments in nonverbal communicative behaviors used in social interaction, ranging, for example, from poorly integrated verbal and nonverbal communication to abnormalities in eye contact and body language or from impairments in understanding and use of gestures to a total lack of facial expression and non-verbal communication. Finally, deficits in developing, maintaining, and understanding relationships, such as difficulties in adjusting behavior in different social contexts, difficulties in pretend play or making friends, and a lack of interest in other people.

The neurocognitive line of studies has shown that infants described as having an increased likelihood of autism show alterations in attention to socially relevant stimuli (6, 14, 25), and that children with ASD have alterations in the development of one of the precursors to the ability to understand the desires, intentions and beliefs of others, i.e., *joint attention* (7, 24, 26). Alterations in face perception, emotional processing and visual scanning of faces have also been described in the literature (7, 14, 16, 27, 28).

One of the most reported difficulties in autism has been the alterations in mentalizing ability, also known as *theory of mind* (ToM) (5–13). Mentalization is a fundamental skill that enables individuals to understand the thoughts and feelings of others, allowing them to make inferences about the mental states of those around them (29–31). This ability involves the capacity to make inferences about states of mind that are not directly observable, ultimately enabling individuals to predict the behavior of others (8, 30, 32). The most reliable evidence of one’s capacity to understand another person’s perspective is their ability to understand ‘false belief’ situations. In these scenarios, an individual predicts or explains someone else’s behavior based on that person’s beliefs, rather than the actual reality as known by the observer. The ability to mentalize is particularly tested when the perceptions of two individuals diverge, and the observer must consider not only what they see or know but also what the other person sees or knows (33). Alterations in the ability to understand and engage in social interactions are often observed in individuals with autism. This includes difficulties in comprehending the social use of language, interpreting the intentions and emotions of others in social situations, and engaging in mentalization processes. Neurobiological research has shed light on the underlying neural mechanisms.

Studies have identified reduced neural responses in key regions of the mentalization brain network, notably the temporoparietal junction (TPJ) and the medial prefrontal cortex (mPFC), during mentalization tasks (10–13). However, it’s essential to note that the mentalization brain network comprises a complex set of neural connections, encompassing not only the TPJ and mPFC but also the precuneus/posterior cingulate cortex, temporal poles, and superior temporal sulcus (7, 34–39). These intricate connections highlight the brain’s role in mentalization processes, enabling individuals to understand and anticipate the actions of others. Furthermore, comparative neuroanatomical evidence has shown that in humans, the brain region including the TPJ and posterior superior temporal sulcus (STS) is significantly expanded compared to non-human primates. This expansion facilitates functional connections with sensory areas associated with body perception (extrastriate body area - EBA), movement (middle temporal area - MT/V5), and attention processes (inferior parietal lobe). These connections are vital for processing social information and exhibit selective responses from the earliest stages of development (40, 41). In addition to the alterations in the TPJ and mPFC, studies on ASD have also reported atypical functioning in the temporal lobe, including the STS (27, 42–48). These findings suggest functional connectivity abnormalities within the mentalization brain network, emphasizing the importance of considering these factors when seeking to understand the core social difficulties in individuals with ASD.

The social nature of the mentalizing ability underscores its relational component, prompting questions about the levels of complexity where this ability is required or involved. For instance, understanding scenarios involving false beliefs, as mentioned earlier, necessitates the capacity to think from another person’s perspective in the context in which the observer (oneself) is involved. Notably, studies have described a specific phenomenon known as ‘camouflage,’ which is observed in both men and women with ASD who present high cognitive abilities. This phenomenon plays a significant role in compensating for communication difficulties and may contribute to the challenges of diagnosing ASD in womenU (49–53). Indeed, almost 25% of the caregivers’ reports of girls with ASD described that the child presents a powerful desire to please others compared to only 10% of the caregivers’ reports of boys with ASD (54). The phenomenon of social camouflage comprises an explicit effort to “mask” or “compensate” autistic characteristics and use conscious or unconscious techniques that result in a behavioral presentation less marked by autistic characteristics (51, 55). More specifically, authors have described making eye contact during conversation, using learned phrases or previously prepared jokes in conversation, imitating the social behavior of others, imitating facial expressions or gestures, and learning and following social scripts as examples of camouflage. While many individuals adjust their behavior based on social expectations or influence, one of the distinctive aspects of camouflage in individuals with ASD is its demand for significant cognitive effort. This effort can be draining and may result in heightened stress responses, including social overload, anxiety, and depression. Moreover, it can negatively impact self-identity development. Unlike typical social adaptation, camouflage in people with ASD involves a unique and often exhausting compensation and masking mechanism (56). A possible interpretation could be that the alterations described in the mentalization network make the adaptation of one’s behavior to social demands so complex and demanding for individuals with ASD that it results in incurring in significant emotional and psychological costs for them.

Taken together, this evidence highlights the relational dimension of social functioning, in terms of a diverse knitting of human relations that could entail emotions, satisfactions (or not) of needs, contexts, etc. Importantly, these relations can be experienced as reciprocal, synchronized, stable, i.e., trustworthy and secure, or unpredictable, ambivalent, non-reciprocal, and even threatening, i.e., unreliable and insecure, from a very early age. Therefore, if the social phenomenon is closely associated with how those interactions are mutually experienced, and autism is described as having social interferences, the question that naturally arises should be does autism impact the relationship between infants and their caregivers? If it does, how does autism impact those relationships?

In the following section, we will delve into the topic of attachment development in autism. We will also examine the neuroscience perspective on attachment in autism to gain a more comprehensive understanding of the condition and its potential treatment with MBT.

## 3 Attachment and autism

Attachment theory has made significant contributions to the fields of psychology, psychopathology, education, and health in recent years. However, studying attachment patterns in relation to autism can be a complex undertaking.

Historically, attachment has been assessed using the Strange Situation Test (57). This method identifies various types of attachment based on a child’s reactions to separation and reunion situations with their caregivers and strangers. However, this type of assessment may not be suitable for children with autism who struggle with changes to their daily routine and find unexpected separations distressing, as noted by (58, 59). Considering these elements, some authors modified the Strange Situation procedure so that the mother or stranger remained with the child throughout the procedure, and the child was never left alone (59–61). Studies showed that when a modified Strange Situation paradigm was used, no significant differences were found between the groups of children with ASD and children with other neurodevelopmental disorders in criteria for proximity, maintenance of contact, avoidance of proximity or contact resistance (60, 61). Moreover, findings showed that mothers of securely attached children with autism scored higher on the sensitivity scale than mothers of insecurely attached children, even when controlling for the children’s level of functioning, their diagnosis, and their level of responsiveness to their mothers (62). This evidence is showing that children with autism can indeed form a sense of security towards their caregivers, and that attachment-related behaviors in autism are linked to the caregiver behaviors, specifically sensitivity and emotional availability (63).

The literature on the topic of attachment in children with autism has produced mixed results. Some studies have found that these children have higher levels of insecure attachment compared to other groups. However, there have been inconsistencies in the analysis and evaluation methods used (64). Recent evidence suggests that children with ASD and intellectual disability may experience more severe behavioral and emotional problems, as well as attachment difficulties, compared to children with other developmental disabilities (65). Additionally, children with ASD tend to have less close attachment relationships and more inhibited attachment behaviors than children with other developmental disabilities. Studies consistently show that children with autism react to their caregiver’s separation, that they direct more social behavior at the caregiver than at a stranger, and their proximity-seeking behavior increases after separation from the caregiver (59). It has been found through studies that caregivers play a significant role in promoting secure attachment in children with autism. Interventions that focus on enhancing maternal/paternal sensitivity and strengthening the parent–child relationship can be helpful in achieving this (59, 62–64). Children with autism and their families may be at increased risk of developing insecure attachment patterns due to difficulties in communication and social functioning associated with autism, and the prevalence of similar traits and mental health problems among family members (16, 64, 66–68). Furthermore, there is evidence that suggests that children with autism and their parents can experience higher levels of stress (69, 70), making attachment security more complex to achieve. In addition, children with autism that also have attachment difficulties present particular challenges for therapists, researchers, and all those seeking to understand their symptoms and provide appropriate support (70). Based on this evidence, interventions aimed at addressing behavioral and emotional problems in children with ASD may benefit from a model that uses attachment relationships to help the child regulate their emotions with the help of caregivers.

During the dyadic interaction between children and their attachment figures, a complex process of accommodation and coordination occurs at multiple levels, resulting in the emergence of self-reflecting awareness and socioemotional skills (71–74). This interaction can be understood as the result of a biologically evolved neural program which aims to organize behavior in times of need, especially in mammals (75, 76). Hence, during this dyadic interaction, brain activity is modulated in both the children and the attachment figure, through psychobiological accommodation, synchronization, and coordination of physiological rhythms (e.g., brain and heart activity) (71, 73, 77–79). Brain structures such as the amygdala, the anterior cingulate insula and orbitofrontal cortices play a major role in these processes (80, 81). A large body of literature has demonstrated the occurrence of synchrony in physiological variables (e.g., skin conductance, respiration, heart rate) during the caregiver-child interaction. Synchrony in brain activity during dyadic interaction is associated with parental sensitivity and positive socio-emotional outcomes (82, 83). In turn, increased level of parental stress is associated with less brain-to-brain synchrony in areas implicated in inferential processes for mental states and social cognition (e.g., prefrontal cortex, dorsolateral prefrontal cortex, and frontal gyrus) (82). Research has shown that synchronicity in cardiac activity and respiration can serve as biomarkers for vagal tone, which is related to physiological regulation and stress, as well as parents’ engagement capacity (84, 85). For example, research has found that there is an increase in mother-infant concordance regarding heart activity during periods of affect and vocal synchrony in dyadic interactions. This is positively correlated to child/family functioning, as noted in the work of Feldman et al. (79).

The neuropeptide oxytocin is one of the biomarkers of attachment. According to research, oxytocin contributes to interpersonal bonding, parental care, trust establishment and social attachment in typical development (86, 87). In autism, research has indicated that intranasal oxytocin can positively impact social functioning and attachment (88, 89).

For example, an experimental trial on multiple-dose oxytocin treatment found a beneficial effect on repetitive behaviors and feelings of avoidance (90). It has been suggested that oxytocin may reduce emotional arousal in the amygdala circuitry (88). The amygdala has many oxytocin receptors and receives direct axonal projections from hypothalamic nuclei (91). This allows for direct neuroendocrine modulation of amygdala-centred circuits, contributing to its role in social processing. Moreover, the amygdala’s distributed connectivity within the social brain provides it with a central position for modulating various brain networks crucial for social cognition. In autism, functional connectivity of the amygdala was found to be significantly attenuated to the bilateral orbitofrontal cortex and the right pSTG, which are both important hubs for social functioning, according to research conducted on young male adults (88). However, the effects of oxytocin are not consistent across all studies. For example, a recent study in boys and girls with autism showed that only the group who received intranasal oxytocin and concurrent intensive psychosocial training demonstrated a notable enhancement in social responsiveness (89).

The above reflects the complex nature of the oxytocin influence on social cognition. Further research is needed to fully understand oxytocin’s mechanisms and potential therapeutic applications in autism.

Based on the reviewed findings, it is important for an integrative psychotherapeutic model for individuals with autism to consider the child’s characteristics, their impact on the bond with their caregiver, and the effects of having a diagnosis. How a caregiver perceives the mental state behind a child’s behavior can affect how they respond, ultimately affecting the quality of their relationship.

This highlights the vital link between attachment and mentalizing, which will be further examined in the following section, particularly concerning autism.

## 4 Attachment and mentalizing in autism from a relational perspective

Studies indicate that a diagnosis of autism can have positive effects on the parent–child relationship in certain cases. Parents report decreased negative evaluations of their child’s behavior following diagnosis and individuals with autism report gaining valuable insights into their past experiences and being able to reframe their sense of self after receiving a diagnosis. This newfound understanding can serve as a protective factor in the relationship between parents and their child with autism (69).

When it comes to self-identity and mentalizing, it’s important to note that the camouflage mechanism can play a role in self-awareness. While many individuals may model their behavior based on societal expectations or influence from others, individuals with autism may use camouflage as a way to compensate and mask their true selves. This can require a significant amount of cognitive effort, leading to heightened stress responses, social overload, anxiety, depression, and even a negative impact on self-identity development [as noted by Hull et al. (56)].

The evidence presented supports the significance of addressing the ability to understand and interpret the thoughts, feelings, and intentions of oneself and others, aligning with the core principles of mentalization-based therapy. In the following sections, we aim to incorporate these elements to propose a therapeutic model capable of helping transform one’s and others’ sense of self.

## 5 Foundations and applications of mentalization-based treatment: exploring adaptations for autism spectrum disorder

MBT is an evidence-based psychotherapy approach with the primary goal of improving individuals’ capacity to comprehend their own thoughts and emotions, as well as those of others. This approach combines concepts from psychoanalysis with attachment theory and research on social cognition. MBT has demonstrated effectiveness in reducing symptoms, enhancing interpersonal skills, and ultimately, elevating overall quality of life. Originally, MBT was developed to foster mentalizing in individuals diagnosed with borderline personality disorder (92). Over time, MBT has evolved to address a broad range of applications for patients with different diagnoses and at different developmental stages. Specialized versions of MBT have been created to attend to the specific needs of adolescents (MBT-A) and children (MBT-C). Additionally, MBT has been modified to address conditions such as eating disorders, depression, post-traumatic stress disorder (PTSD), and antisocial personality disorder (93).

The framework of MBT is built on two key assumptions. First, it believes that the ability to understand mental states is developed through early attachment relationships and is closely intertwined with the development of the self. Second, MBT recognizes that disruptions in these early attachment relationships can hinder the growth of an individual’s capacity for mentalization and the development of their self-structure.

MBT mainly proposes a developmental model of the self, drawing from concepts in developmental psychology, attachment theory and psychoanalysis. However, as previously mentioned, when adapting MBT for individuals with ASD, it’s crucial to establish a strong foundation rooted in neuroscience-based evidence while coherently incorporating attachment theory and mentalization processes. To address this challenge, the following sections will explore the therapeutic principles and applications of MBT, with a focus on the model for psychosis as a starting point. Additionally, we will delve into the current state of MBT in the context of autism.

### 5.1 MBT in psychosis

The primary objective of MBT is to establish an intersubjective narrative construction space that fosters the development of mentalizing capacity, enabling individuals to effectively process emerging thoughts and feelings. This is achieved by establishing a patient-therapist relationship that evokes affective states and engages the patient in a reflective process. An essential component of MBT is the repair of mentalizing ruptures that may occur during therapy sessions.

In psychosis, Debbané et al. (20) have proposed that MBT can be effectively applied in the treatment of young people at risk for psychosis by adhering to three clinical principles. The first principle involves adopting a therapeutic stance that fosters mentalizing by stimulating curiosity about the complexity of mental states. The therapist plays an active role in encouraging reflection on interpersonal experiences, including the therapy session itself. The second principle focuses on affective experiences and encourages patients to recognize and verbalize their feelings while reflecting on the events that preceded them. This promotes a trusting relationship and enables the patient to reconceive non-mentalizing explanations of behavior in terms of opaque and complex states of mind. The third principle centers on enhancing embodied mentalizing, where the therapist supports patients in finding words to express affective states and links them to bodily or perceptive experiences, thus enhancing their sense of self-continuity and facilitating a coherent view of their self-experience. The primary focus of the therapist in MBT is to make affects the main topic of joint attention during therapy sessions. By increasing awareness of their own and others’ minds, young individuals who are at risk of developing psychosis can use their thoughts to regulate and communicate their affective experiences (94). The therapist helps the patient recognize and verbalize their emotions, as well as reflect on their interpersonal experiences. This can lead to a stronger sense of self and help individuals regulate and communicate their emotions more effectively. For individuals with psychosis, MBT can be particularly beneficial because it focuses on increasing their awareness of their own and others’ minds, helping them restructure their thinking patterns towards more flexible and reality-based beliefs. In general, therapeutic approaches that prioritize mentalizing can assist individuals in maintaining their resilience against fixed and distorted thought patterns, even when they are at risk for psychosis due to neurogenetic or other factors (95).

Based upon these considerations about the MBT model in psychosis, and considering the difficulties described in mentalizing ability in autism, may the question arise as to whether it is possible to develop an autism MBT model that integrates neuroscience, attachment, and mentalizing evidence?

### 5.2 Social functioning in psychosis and autism

While there are distinct neurodevelopmental characteristics between autism and psychosis, social functioning challenges have also been observed in individuals with psychosis, particularly those diagnosed with Schizophrenia Spectrum Disorders (SSD) as a proxy for psychosis (1–3, 96, 97). Research suggests that individuals with schizophrenia often struggle with social cognition, including difficulties inferring others’ intentions (i.e., mentalization, (1–4) which is also an element in autism).

Social cognition entails various cognitive processes that integrate different brain structures and networks (1, 34) and allows individuals to understand the thoughts, intentions, beliefs, and feelings of other people (1, 35, 98). Moreover, social cognition underlies social behavior and enables functioning in social contexts (1, 34, 36) because those cognitive processes embed information about other persons and about interpersonal norms and procedures to participate efficiently in the social world (98). As in the case of autism, evidence in SSD has shown that mentalizing impairment has been associated with an abnormal activation in brain regions related to the mentalizing network during mentalization tasks, i.e., the medial prefrontal cortex (mPFC) and the temporoparietal junction (TPJ) (2). Moreover, in interplaying games, patients with schizophrenia showed an opposite pattern of bargaining compared to control individuals, which was in association with brain regions that are related to social decision mechanisms, i.e., the mPFC, the inferior parietal lobule, and the TPJ (35).

Unlike psychosis and schizophrenia, the distinction of ASD as a neurodevelopmental condition presents a significant opportunity to explore the challenges in understanding mental states. This difference can be crucial for developing a specialized MBT model tailored for autism. It is possible to consider that the pervasive feature of autism encompasses an early and primary interference with the ability to mentalize from the beginning. Thus, mentalization-based interventions may be beneficial for individuals on the autism spectrum who struggle to identify and distinguish between different mental states in themselves and others. This is because, through MBT, individuals can develop a more nuanced awareness of their thoughts, emotions, and motivations, leading to increased self-reflection and self-understanding. Moreover, considering that MBT recognizes the interactive nature of mentalizing and that mentalization deficits are associated with social dysfunction (3, 7, 99), a therapy focusing on enhancing the ability to mentalize both oneself and others, can foster improved interpersonal understanding and communication.

### 5.3 Current studies on MBT for children with ASD

Current interventions to improve social and mentalizing abilities in individuals with ASD have been mainly based on Cognitive Behavioral Therapy (CBT) (100, 101). However, during the last years, the idea of incorporating therapeutic strategies specially focused on improving mentalizing abilities (e.g., MBT) for individuals with ASD has become more accepted (102–105). In addition, studies (103, 106, 107) have preliminary assessed the effectiveness of these types of interventions by adapting ideas from MBT (31, 108, 109), showing an incipient development of therapeutic strategies with MBT as theoretical background.

Studies have shown the benefits of working alongside parents and focusing on relational aspects.

It has been argued elsewhere the potential of the MBT-C model to increase the child’s capacity for emotional regulation and improve general psychosocial functioning (102). This is achieved using a scaffolding approach that provides a secure and predictable therapeutic framework and by working with parents in a therapeutic manner that models a more connected interactive style. It is suggested that using MBT-C, it can be possible to work on a new model of relationship that fits the child’s “regulation profile” and the caregiver’s capacity to learn and apply a new way of connecting and communicating (102). Moreover, there is evidence that showed the impact of an MBT group intervention for parents of children with autism associated with an improvement in parental reflective functioning and emotional regulation and a significant interaction effect between the time of intervention and parents’ sense of efficacy (107).

Additionally, there are interventions that may not specifically focus on mentalizing, but they still work towards improving related capacities. These interventions can offer valuable information on the potential effectiveness of this model for the targeted population. In this regard, there is evidence that children with autism improve their social communication skills by increasing their role as initiators of social interactions such as improvements in social and emotional behavior, communication, eye contact, joint attention, and imitative play (110).

It has been also found that working with children with ASD through interactions between children and parents using a Developmental, Individual Difference, Relationship-Based model of intervention (DIR) may enhance important aspects of mentalizing such as joint attention and regulation, engagement across a wide range of emotions, two-way communication, and complex social problem solving (111).

The evidence examined thus far suggests that treatments centred around mentalizing and related skills have the potential to significantly improve social functioning, psychological adjustment, and emotional regulation in children with autism. However, there is currently no research supporting the effectiveness of a MBT model specifically for children with ASD.

In this regard, we argue that in order to develop therapeutic approaches in autism suitable to be empirically tested, there is a need for a more robust underlying theory. We propose that such a theory should integrate attachment, neuroscience, and mentalizing. In this article, we have presented some of the current advancements in autism research, specifically in the areas of attachment and mentalizing, from a neuroscience perspective.

In the subsequent section, we outline possible avenues for progress, considering both clinical and research possibilities.

## 6 Working from a mentalization-based approach: a proposal of clinical strategies for the work with children with autism

After reviewing the available evidence, we propose a set of elements that should be considered within current mentalization-based models in order to better meet the needs of children with ASD.

By focusing on a core capacity that may promote resilience in a wide range of children with various presenting problems, MBT aims to be a transdiagnostic therapy that can be adapted to the particular needs of a range of difficulties.

Regarding ASD, MBT approaches, and especially those that work with children (e.g., MBT-C) may be helpful as they offer an alternative model of the relationship between the caregiver and the child, which fits both the child’s and caregiver’s capacities to reflect on their own and other’s mental states and generalize a new way of regulating, connecting, and communicating with themselves and others. MBT with children may support developmental experiences through a secure, predictable, yet flexible therapeutic framework to improve psycho-social functioning and increase emotional regulation skills.

Considering these elements, we propose a body of therapeutic actions that are focused on the complex interplay among the therapist, the child, and the caregiver in the context of ASD. In this respect, it is important to note that ASD exhibits a widely heterogeneous range of social and cognitive symptoms, which has challenged comprehension and therapeutic approaches. It has been argued that this enormous phenotypic heterogeneity is closely related to a complex multifactorial etiology (112–116), making even more complex the understanding of this neurodevelopmental condition. Importantly to the conceptualization of therapeutic strategies of the social challenges present in ASD is to notice that autism does not necessarily co-occur with intellectual disability. Indeed, it has been described that the comorbidity with intellectual disability is around 33.0% (117). Furthermore, clinical therapeutic approaches in ASD should consider that even though communication difficulties are present in both verbal and non-verbal individuals with ASD (4), children with ASD with both cognitive and language difficulties exhibit different challenges to address. This is particularly important for both pre-verbal children and children with language or cognitive difficulties. These individual characteristics should be taken into account, irrespective of the age, in order to adapt each clinical strategy to each level of development of each child and their unique challenges. Based on these considerations, our proposal includes two key aspects: (1) the specific developmental obstacles that the child may face, regardless of their age, and (2) the work with parental mentalizing ability to enhance the caregiver’s ability to understand the child’s communicative challenges.

a) Knowing the features of autism while keeping the not-knowing stance: a comprehensive understanding of relevant information and scientific knowledge will contribute to debunking myths and misconceptions regarding autism and individuals with ASD. This knowledge holds significant potential in developing self-identity and fostering a deeper sense of self-understanding by:

- Acknowledging the child’s unique experience and co-constructing the diagnosis together—not taking the child’s experience for granted.
- Being curious about their states of mind that arises in situations linked with difficulties in social communication and/or related with their pattern of interests and behaviors.

Considering the presence of restrictive and repetitive behaviors and patterns, it is crucial to assist the child in balancing an “overinterpretation” and an “overlook” of both their experiences and their own characteristics. The exercise of weighing each individual’s role in a situation (whichever may be) is challenging. In addition, social interactions are highly complex situations that require predictive abilities, and mentalization is a critical skill needed to interpret, comprehend, and attribute both one’s own and other’s behaviors (7, 35, 118–120). The presence of a diagnosis can add some confusion to this exercise. It is possible that the child becomes lost in the details of the attributions or confused, unravelling the net of social thoughts and interpretations. “Overinterpretation” and “overlook” pathways can both interfere with the awareness of self-in-relation-to-other and the self-in-relation-to-the-world (95). Considering the child’s development stage, this co-constructing experience between the therapist and the child can contribute to restructuring (re-routing) the functional configuration of thinking towards more flexible patterns (95).

b) Creating mentalizing narratives about events related to social communication difficulties: it may be helpful for children to have someone join them in reflecting on and understanding their thoughts and feelings about their own unique (and in some cases, “socially uncommon”) characteristics of personality, interests or thoughts in a curious rather than critical approach. Exploring autism’s meanings constructed from non-mentalizing interactions with others plays a crucial role in self-identity. For example, health professionals, teachers, classmates, relatives, etc. may give misleading labels, leading to children feeling confused, misunderstood and invalidated about themselves and their experiences.
c) Working with the autobiographical narrative: creating a life story that incorporates the child’s own characteristics, interests and experiences, can strengthen self-esteem, emotional regulation and self-identity. By exploring the impact of cultural perspective regarding autism (e.g., stigma or gender bias) in the autobiographical narrative of the child, self-awareness, re-routing and mentalization are reinforced.
d) Working with process rather than content: learn and understand in the here-and-now how to cope with and regulate the emotions associated with rigid thinking and/or restrictive interests and behaviors, the presence of comorbidities or symptoms, sensorial interferences, among others.
e) Working with parents (caregivers) in mentalizing autism: it may be helpful to visit specific episodes where the child-caregiver dyad faces autism-related issues and to mentalize what happened. The parent-therapist work should reflect on the caregiver’s thoughts and feelings related to the child’s characteristics and behaviors in order to revise the role of the diagnostic on their relationship with their child. For example, a diagnosis may be overused when it is used excessively to explain a child’s behavior, disregarding other possible causes and hindering a complete comprehension of the child’s inner thoughts and feelings. Also, mentalizing the diagnosis can promote an empathic awareness of when the caregiver’s own anxieties lead them to intrusive and controlling behaviors in the relationship with the child. On this point, it is also important to consider that relatives of children with autism might display subclinical symptoms (16), so the parents’ self-identity may also be questioned in the psychotherapeutic process, which may add to the challenge of mentalizing their children’s experiences, especially if the caregiver has not yet had the opportunity to reflect on their own childhood experiences.
f) Collaborative work with educational and health systems: therapeutic strategies should include collaborative work with the child’s educational system. Communication with the school regarding the child’s coping at school and how to support them is crucial to reinforce their self-identity development and facilitate the inclusion of the child in the social context. Additionally, and considering the eventual presence of comorbidities, therapeutic strategies should also include working together with the health system, such as a psychiatrist, neurologist, occupational therapist, and speech therapist, in order to strengthen the interventions and to understand the entire network of meanings in which the child is immersed.

## 7 Advancing research: practical guidance and prospects

As we mentioned earlier, no research currently supports the effectiveness of a MBT model specifically for children with ASD. We have argued that a therapeutic approach worthy of empirical testing needs a more robust underlying theory. Here, we have presented some of the current advancements in research in ASD, specifically in the areas of attachment and mentalizing, from a neuroscience perspective. Although there have been some developments in this area, more information is needed to understand how therapeutic strategies aiming to enhance mentalizing might work better for individuals with ASD.

Therefore, to move forward in the research field, it is crucial to conduct further research exploring the neurobiological mechanisms underlying attachment and mentalizing, particularly in the context of ASD.

Once equipped with this knowledge, the subsequent step may involve the design of therapeutic approaches that seamlessly integrate attachment theory, neuroscience, and mentalizing. Such integration has the potential to give rise to tailored therapeutic approaches that take into account the distinctive neurobiological characteristics of individuals with autism, potentially resulting in enhanced care and the facilitation of empirically validated interventions.

Following this, the research should shift its focus to the conduct of empirical studies aimed at evaluating the effectiveness of these therapeutic approaches. These studies may encompass a range of methodologies, including randomized controlled trials, feasibility studies, and secondary analyses. Therapeutic outcomes should include improving social functioning, psychological adjustment, and emotional regulation (in addition to mentalizing abilities). Additionally, achieving these improvements may reduce caregivers’ distress, which can be seen as a secondary therapeutic outcome.

To assess these therapeutic outcomes, studies might benefit from the incorporation of measures of general social functioning, social interaction functioning, communication abilities, and the presence of restricted, repetitive, and stereotyped behavior. For example, studies can use validated scales such as The Social Communication Questionnaire (SCQ) (121–123). For the assessment of mentalizing in children, the Reflective Functioning Scale (124, 125) can be used to score mentalizing in the Child Attachment Interview (126), and also in the Parent Development Interview (127) for the assessment of mentalizing in parents. In this context, it may also be beneficial to evaluate social camouflage (for example, using the CAT-Q) (55). Since social camouflage involves using mechanisms to make specific features of personality or behaviors appear “less autistic,” it is possible for an individual with ASD to interpret their actions and personal traits by considering how others might interpret those features. Although this process of interpretations and attributions may be iatrogenic for the individual, it involves a complex mentalization process. It is important to note that the consequences of the use of masking mechanisms of “autistic” traits should be addressed in the therapeutic context, due to the negative impact that it could have on self-esteem and self-identity.

Finally, it is crucial to acknowledge the heterogeneous nature of autism, which is reflected in important individual differences among individuals with ASD. This includes considering factors such as age, gender, language abilities, cognitive performance and cultural backgrounds. It is equally important to consider potential cultural differences that may impact outcomes in different populations and situations when examining the effectiveness of MBT for individuals with autism.

## 8 Conclusion

In this paper we have shown that comprehensive models to understand the psychological processes of children with ASD have prospects for advancement. Integrating attachment, neuroscience, and mentalizing can help in achieving this goal. While there is room for improvement, there is already a path to follow. The current evidence supports the importance of utilizing neuroscience within psychological therapeutic models like MBT. Our literature review suggests that MBT models are suitable for integrating the latest neuroscientific findings into therapeutic considerations. This approach will provide a secure environment for individuals with autism to address any psychological issues they may be experiencing—particularly those related to mentalizing.

MBT has the potential to be a valuable therapeutic approach for children with ASD. By fostering mentalization, it can help children develop a more nuanced awareness of their thoughts, emotions, and motivations, ultimately leading to increased self-reflection and self-understanding. Furthermore, MBT promotes the development of more flexible thinking patterns, aiding individuals in coping with and regulating their emotions effectively.

We also highlighted the importance of considering the caregiver’s role in the therapeutic process. Collaborative work with parents or caregivers can enhance their understanding of the child’s unique experiences and challenges, ultimately strengthening the parent–child relationship. This approach extends to educational and health systems, emphasizing the need for cooperation to support the child’s social inclusion and overall well-being.

We additionally emphasize the importance of recognizing the diverse needs of pre-verbal children and those with language or cognitive difficulties as a crucial aspect to consider when tailoring effective clinical strategies. It is important to acknowledge individual characteristics and adapt interventions accordingly, regardless of age. Proper therapeutic strategies must take into account the stage of development at which each child is and should be adapted to address the challenges specific to that developmental level. We argue that an appropriate therapeutic approach should not only foster a supportive environment for the child’s development but also a caregiver-child relationship.

However, the next step is to conduct studies that evaluate the outcomes of MBT interventions, considering factors such as social functioning, psychological adjustment, emotional regulation, and mentalizing abilities. These studies should be mindful of the heterogeneity within the autism spectrum, accounting for variations in age, gender, language abilities, cognitive performance, and cultural backgrounds.

In summary, we offer a promising avenue for the development of effective therapeutic approaches for children with ASD. By incorporating attachment theory, neuroscience, and mentalization processes, we can work toward providing tailored interventions that address the unique needs of each child. Through empirical research and a collaborative effort involving caregivers, educational systems, and healthcare professionals, we can advance our understanding and application of MBT, ultimately improving the well-being and social integration of children with autism.

## Data availability statement

The original contributions presented in the study are included in the article/supplementary material, further inquiries can be directed to the corresponding authors.

## Author contributions

SC-C: Conceptualization, Writing – original draft, Writing – review & editing. PS-I: Conceptualization, Writing – original draft, Writing – review & editing. KB: Conceptualization, Writing – original draft. AG-C: Writing – original draft. NM: Conceptualization, Writing – review & editing.
